# CK2 and PI_3_K are direct molecular targets of quercetin in chronic lymphocytic leukaemia

**DOI:** 10.18632/oncotarget.17246

**Published:** 2017-04-19

**Authors:** Maria Russo, Alfonsina Milito, Carmela Spagnuolo, Virginia Carbone, Anders Rosén, Paola Minasi, Fabio Lauria, Gian Luigi Russo

**Affiliations:** ^1^ Institute of Food Sciences, National Research Council, Avellino, Italy; ^2^ Department of Clinical and Experimental Medicine, Division of Cell Biology, Linköping University, Linköping, Sweden; ^3^ Current address: Stazione Zoologica “Anton Dohrn”, Villa Comunale, Napoli, Italy

**Keywords:** quercetin, protein kinase CK2, PI_3_K, chronic lymphocytic leukaemia, Mcl-1

## Abstract

Despite the encouraging results of the innovative therapeutic treatments, complete remission is uncommon in patients affected by chronic lymphocytic leukaemia, which remains an essentially incurable disease. Recently, clinical trials based on BH3-mimetic drugs showed positive outcomes in subjects with poor prognostic features. However, resistance to treatments occurs in a significant number of patients. We previously reported that the multi-kinase inhibitor quercetin, a natural flavonol, restores sensitivity to ABT-737, a BH3-mimetic compound, in both leukemic cell lines and B-cells isolated from patients. To identify the molecular target of quercetin, we employed a new cell line, HG3, obtained by immortalization of B-cells from a chronic lymphocytic leukaemia patient at the later stage of disease. We confirmed that quercetin in association with ABT-737 synergistically enhances apoptosis in HG3 (combination index < 1 for all fractions affected). We also reported that the cellular uptake of quercetin is extremely rapid, with an intracellular concentration of about 38.5 ng/10^6^ cells, after treatment with 25 μM for 5 min. We demonstrated that the activity of protein kinase CK2, which positively triggers PI_3_K/Akt pathway by inactivating PTEN phosphatase, is inhibited by quercetin immediately after its addition to HG3 cells (0–2 min). PI_3_K activity was also inhibited by quercetin within 60 min from the treatment. The combined inhibition of CK2 and PI_3_K kinase activities by quercetin restored ABT-737 sensitivity and increased lethality in human leukemia cells.

## INTRODUCTION

According with the recently published statistics on cancer in USA, B cell-chronic lymphocytic leukemia (CLL) remains the most common form of adult leukemia in the Western world, with 18,960 new cases expected in 2016 and 4,660 estimated death [1]. CLL is defined a malignant lymphoproliferative disorder of mature clonal B lymphocytes (B-CLL) that accumulate in the blood and other lymphoid tissues. The diagnosis of CLL occurs when B-CLL count is > 5,000/μL. Other features of the disease are established and periodically reviewed by the international working group of CLL (iwCLL) [2, 3]. Although the origin of B-CLL is still controversial, recent evidence suggest that genetic and epigenetic alterations occurring in pluripotent haematopoietic stem cells (HSCs) in the earliest phase of B lymphocytes may possibly lead to CLL [2, 4]. According to this model, antigenic stimulation of CLL HSCs may lead to selection and expansion of mature B cells, with the generation of oligoclonal populations. Additional genetic and epigenetic changes, as well as micro-environmental factors and BCR (B cell receptor) stimulation generate the population of cells which are currently considered the precursor of CLL, i.e., monoclonal B cell lymphocytosis (MBL) [5, 2]. CLL is classified into two major subgroups, in relation to the presence or absence of mutations in the Ig heavy chain variable region (IGHV) genes, to whom correspond different pathogenesis and prognosis. The IGHV-mutated (IGHV-M) forms of CLL are less aggressive with a median survival of 20 years, respect to the 8 years for the IGHV-unmutated (IGHV-UM) CLL [6, 7]. However, despite the importance of the IGHV mutational status as prognostic index of the disease, its determination is expensive and not always available in routine laboratories. These practical difficulties have been partially bypassed by the introduction of alternative biomarkers, such as the expression of Zap70 and CD38, both easily detectable by flow cytometry. The tyrosine kinase Zap70 is involved in T-cell receptor signaling and regulation, while CD38 is a surface antigen. Both markers are highly expressed in IGHV-UM CLL and their over-expression is associated with poor diagnosis and shorter time to treatment and survival, although the correlation with the aggressiveness of the disease and resistance to therapy is more pronounced for CLL expressing Zap-70, than for those with high level of CD38 whose expression vary over time [8, 9].

In the recent years, the efficacy of therapeutic treatments against CLL significantly improved. Currently, in patients with advanced stage disease (Rai III and IV or Binet C) [10, 11], the combination immunochemotherapy consisting of fludarabine, cyclophosphamide and the anti-CD20 monoclonal antibody rituximab (FC-R protocol) resulted in an ORR (overall response rate) of 95%, CR (complete response) rates of 72%, a higher eradication of minimal residual disease and longer duration of response [12, 13]. Unfortunately, despite these significant therapeutic improvements, complete remission remains rare and treatment failure rate is still high, especially in fludarabine-refractory patients and in those carrying TP53 inactivation [8, 14].

Searching for novel treatments, the interest of several groups in the last 10–15 years focused on the possibility to re-establish apoptosis sensitivity in CLL [15–17]. In general, the strategy consists in inhibiting the activity, or lowering the expression of anti-apoptotic factors, mainly belonging to the Bcl-2 and IAP families, which are often over-expressed in CLL as a consequence of the constitutive activation of pro-survival pathways [15]. Among the several natural and synthetic compounds tested, those belonging to so-called group of “BH3-mimetic” deserved particular attention [18, 19]. This term indicates small compounds able to “mimic” the high binding affinity of BH3-only proteins, such as Bim, Bid, PUMA, NOXA, which are natural ligands and inhibitors of pro-survival Bcl-2 family members, which sequester pro-apoptotic Bax and Bak factors. As a consequence of the binding between BH3-only agents and anti-apoptotic Bcl-2 factors, Bax and Bak are released, oligomerize and form pores into the mitochondrial outer membrane. Free cytochrome c activates caspases and induces apoptosis [20, 21]. ABT-737 can be considered the founder of this class of pharmacological agents [22]. It binds with high affinity to Bcl-2, Bcl-X_L_ and Bcl-w, but does not antagonize other anti-apoptotic members, such as Mcl-1 or Bfl-1/A1, which determine resistance to ABT-737 in CLL [23]. The [Mcl-1 + Bfl-1]/Bcl-2 ratio has been validated in a panel of leukemic cell lines as an index to predict the response of CLL to ABT-737 [24]. The orally available analog of ABT-737, i.e. ABT-263 (Navitoclax), showed promising activity in a phase I study with a partial response rate of 35% in patients with relapsed/refractory CLL. However, the frequent dose-dependent thrombocytopenia induced by ABT-263, due to its capacity to inhibit Bcl-X_L_, a key survival protein in platelets, represented a limit to its pharmacological application [25]. A new derivative, ABT-199 (Venetoclax), specifically binds to Bcl-2, instead of multiple Bcl-2 family members, avoiding the Navitoclax's adverse effects [26]. In a phase I clinical study in patients with relapsed or refractory CLL or small lymphocytic lymphoma, response rates ranged from 71 to 79% in subjects with resistance to fludarabine, chromosome 17p deletions, and IGHV-UM. Complete remissions occurred in 20% of the patients [27, 28].

The mechanisms responsible for the over-expression of antiapoptotic Bcl-2 factors in CLL are still unclear. At least in the case of patients carrying del13q14, the explanation may reside in the deletion of microRNAs miR-15a and miR-16, which down-regulate Bcl-2 expression [29]. The redundancy of the Bcl-2 family members and the high affinity of BH3-mimetics only for specific anti-apoptotic factors generate resistance in CLL. As an example, we and others demonstrated that Mcl-1 over-expression confers resistance to ABT-737 and its down-regulation increases ABT-737 lethality in human leukemia cells and CLL [23, 30–32]. Similarly, overexpressed Mcl-1 can also be responsible for ABT-263 and ABT-199 resistance in CLL cells. In fact, high levels of Mcl-1 inversely correlated with treatment response in the phase I study of ABT-263 [25] and protected hematological malignancies from ABT-199 [33, 34].

Based on these evidence, we demonstrated that bypassing Mcl-1 mediated resistance, represents a useful strategy to sensitize leukemic cell lines and CLL to apoptosis. In fact, the combined treatment with quercetin and several apoptotic inducers, including fludarabine and death ligands (e.g., recombinant TRAIL and anti-CD95) resulted in increased levels of cell death [35–38]. Quercetin (3,3′,4′,5,7-pentahydroxyflavone) is a natural flavonoid, subclass flavonol, widely present in fruits and beverages. It is a functionally pleiotropic molecule with multiple potential anticancer properties [39–41]. In the case of Mcl-1, we demonstrated that quercetin can down-regulate Mcl-1 acting on its mRNA stability and protein degradation, but can also inhibit the PI_3_K-Akt pathway, which leads to Mcl-1 activation [31, 32, 42]. When associated with ABT-737, quercetin synergistically induced apoptosis in B-cells isolated from CLL patients and in five leukemic cell lines, as demonstrated by the calculation of the Combination Index [31].

Quercetin belongs to the wide group of natural, anticancer compounds proposed as apoptotic inducers in chemotherapy or in adjuvant chemotherapy when associated with other drugs [15, 43, 44]. Here, we identified in protein kinase CK2 the primary and direct target of quercetin in HG3 cells, derived from a CLL clone immortalized by EBV infection [45]. This cell line was obtained from a patient with IGVH-UM phenotype and biallelic 13q14 deletions with genomic loss of DLEU7, miR15a/miR16–1, resembling the poor prognostic CLL patients. Moreover, HG3 is the first cell line that resembles human B1 cells with regard to surface marker profile (CD5+CD20+CD27+CD43+) and spontaneous Ab-secretion [45].

CK2 is a constitutively active dual specificity kinase that phosphorylates serine/threonine and tyrosine residues with multiple functions in normal and malignant cells (reviewed in [46, 47]). CK2 is composed of two catalytic (α and/or α′) and two regulatory (β) subunits, present in the cells and differently associated to form tetrameric complexes (α_2_β_2_, α′_2_β_2_, or α′αβ_2_), or as single monomers. CK2 phosphorylates multiple physiological and non-physiological substrates, characterized by the presence of a highly specific consensus site, i.e., a region containing acidic residues surrounding the phosphor-acceptor amino acid [46]. CK2 has been identified as a player in the pathogenesis of hematopoietic tumors including CLL [48, 49]. Firstly, elevated levels of CK2 β-subunit phosphorylated on Ser209 have been detected in B-cells isolated from 44 CLL patients [50]. It is worthwhile to note that Ser209 phosphorylation can positively regulate the activity of the holoenzyme contributing to its stability [47]. Secondly, inhibition of CK2 activity resulted in reduced phosphorylation of PTEN (phosphatase and tensin homolog) at Ser380 and of Akt at Ser473 and was associated to apoptosis. Similarly, when both CK2 and PI_3_K inhibitors where combined, cytotoxicity of B-cells increased. The authors concluded that CK2, by phosphorylating, *bona fide*, PTEN on Ser380 blocks it capacity to convert PIP_3_ into PIP_2_ maintaining fully active the pro-survival and anti-apoptotic PI_3_K/Akt pathway. Inhibition of CK2 can rescue PTEN activity increasing apoptosis in CLL [50]. This scenario was confirmed by a parallel work showing that CK2 was overexpressed and hyperactive in CLL and its inhibition correlated with increased PTEN activity and inactivation of PKC, a PI_3_K target. Importantly, the same authors reported that the cytotoxicity of CK2 inhibitors (TBBz, DRB and CX-4945) was minimal on normal T and B lymphocytes, while B-cells isolated from advanced stage (Binet B or C) of CLL patients showed a much higher sensitivity to these drugs [51, 52]. It is worthwhile to note that CX-4945, a potent and selective orally bioavailable inhibitor of CK2, alone [53] or in association with fludarabine [54], induced apoptosis in CLL. CX-4945 is capable to attenuate the PI_3_K/Akt signaling by dephosphorylating Akt on the CK2-specific Ser129 site and on the canonical Ser473 and Thr308 regulatory sites [55].

In the present work, we demonstrate that quercetin directly inhibits CK2 and PI_3_K enzymatic activities in a CLL-derived cell line. This mechanism contributes to restore ABT-737 sensitivity and increase lethality in human leukemia cells.

## RESULTS

### Synergistic effect of quercetin associated with ABT-737 in HG3 cell line

**Figure 1:**
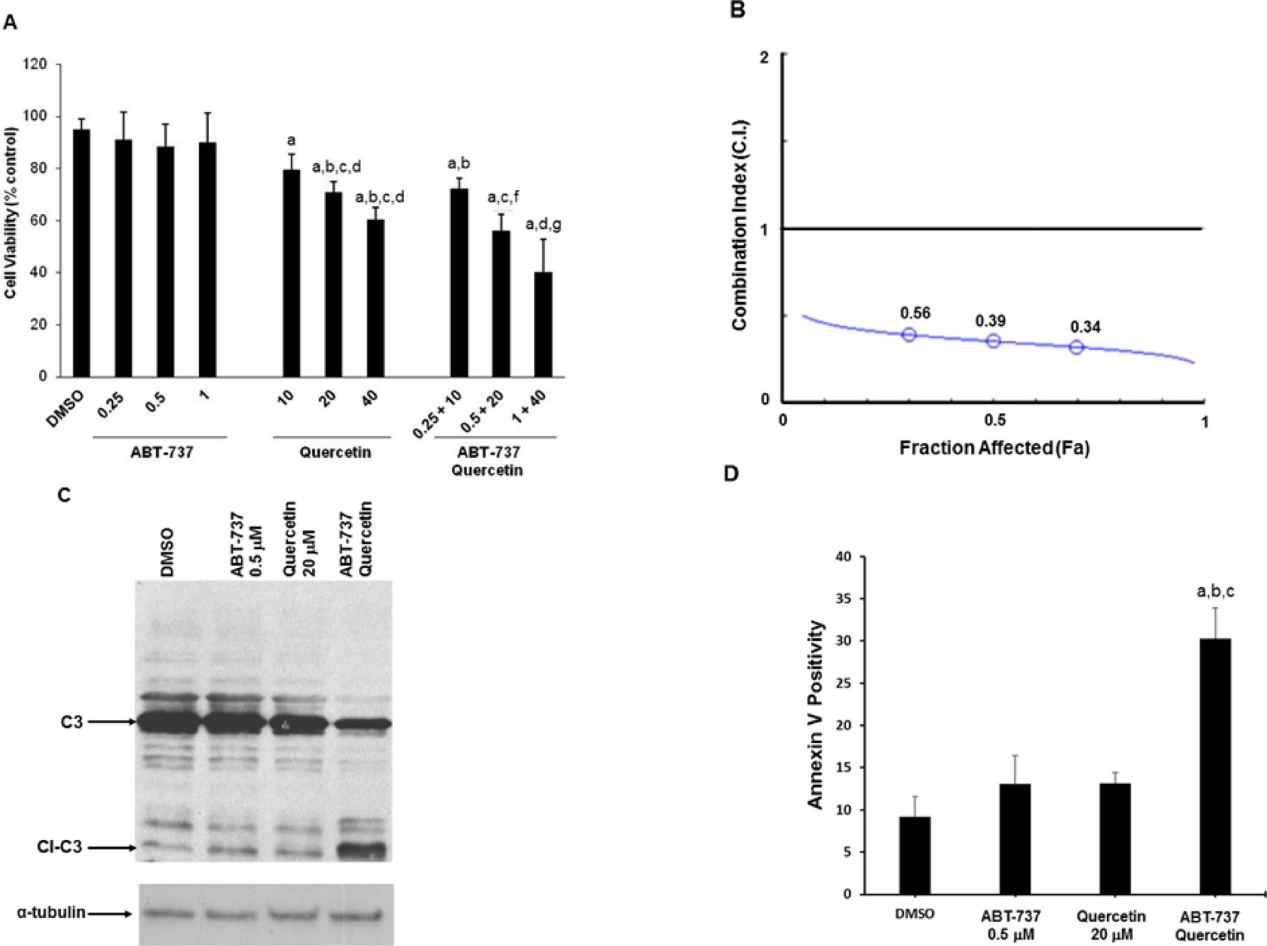
Quercetin in association with ABT-737 induces apoptosis in HG3 cells (**A**) Cells (0.5 × 10^6^/ml) were treated with different doses of ABT-737, quercetin or their combination as indicated for 24 h. Cell death, measured by neutral red assay, is reported as percentage of DMSO (0.1% v/v) treated cells, as described in Materials and Methods. Symbols (a, b, c, d, f, g) indicate significance with respect to DMSO (a) and treated cells (b = 0.25 μM ABT-737; c = 0.5 μM ABT-737; d = 1 μM ABT-737; e = 10 μM quercetin; f = 20 μM quercetin, g = 40 μM quercetin); *p* < 0.001 for all determinations except for a versus e, where *p <* 0.05 (one-way ANOVA test). (**B**) Combination Index (C.I.) isobologram. C.I. values, obtained from neutral red experiment (panel A) using a 1:40 concentration ratio of ABT-737 and quercetin, were plotted against the fraction affected (Fa). (**C**) Proteolytic activation of caspase-3 was measured after 6 h of incubation with the indicated concentrations of ABT-737 and quercetin and their combination. Immunoblot was performed using a specific antibody against caspase-3 (C3 = caspase-3; Cl-C3 = cleaved caspase-3). (**D**) Annexin V measurement in HG3 cells after 18 h incubation with quercetin (20 μM), ABT-737 (0.5 μM) and their combination, as described in Materials and Methods. Symbols (a, b, c) indicate significance; *p* < 0.001 with respect to DMSO (a) and treated cells (b = 0.5 μM ABT-737; c = 20 μM quercetin; d = ABT-737 + quercetin) (one-way ANOVA test).

We previously demonstrated that quercetin is able to restore sensitivity to apoptosis in leukemic cell lines and B-cells from CLL patients when associated with ABT-737 [31]. We verified if this effect was confirmed in HG3 cells. Figure 1A shows that this cell line was resistant to increasing concentration of ABT-737 (0.25–1 μM) and slightly sensitive to 10–30 μM quercetin with a significant cytotoxicity ranging between 20–30%. When quercetin and ABT-737 were associated, the cytotoxic effect increased to about 30, 40 and 60% at the indicated combinations (Figure 1A). The concentrations of quercetin applied have been selected based on previous studies [31] and with the purpose to limit its toxicity in HG3 cells. The combined treatment was synergistic as demonstrated by the calculation of the Combination Index (C.I.) which resulted < 1 for all the fraction affected (Fa). The isobologram in Figure 1B has been extrapolated using a constant ratio between the concentration of quercetin and ABT-737 (i.e., 40:1). The cytotoxic effect of the combined treatment was associated with the activation of an apoptotic process, as demonstrated by caspase-3 cleavage (Figure 1C) and Annexin-V exposure (Figure 1D). Values of caspase-3 activation and Annexin-V exposure increased time-dependently and picked at 6 and 16–18 h, respectively (data not shown).

### Quercetin inhibits the PI_3_K-Akt-Mcl-1 pathway

**Figure 2:**
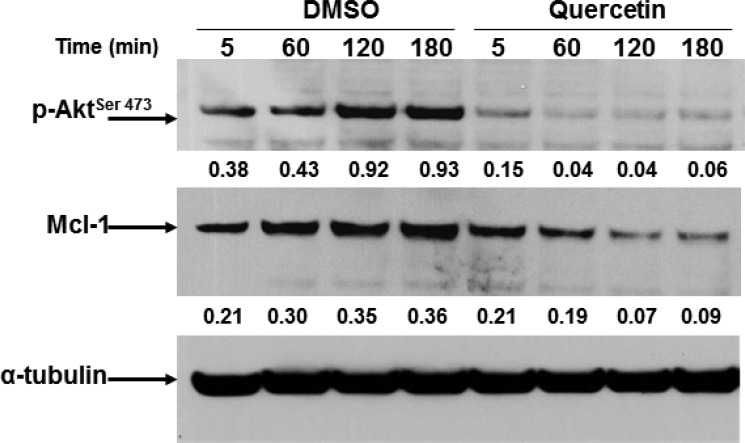
Quercetin down-regulates Mcl-1 and inhibits Akt phosphorylation in HG3 Cells (0.5 × 10^6^/ml) were treated for the indicated time (min) with quercetin (25 μM) or DMSO (0.1% v/v). Immunoblots were incubated for 16 h at 4°C with anti-phospho-Akt (pAkt) antibody (upper panel), stripped and re-probed with anti-Mcl-1 antibody (lower panel). Densitometric analyses were obtained measuring optical density of bands normalized respect to the expression of α-tubulin (numbers below top and middle panels). Immunoblots are representative of at least four independent experiments.

We previously reported the capacity of quercetin to sensitize leukemic cells to apoptosis inducing Mcl-1 degradation [31, 32, 38]. In addition, it is well known that Mcl-1 is activated by multiple pathways in CLL, including PI_3_K/Akt signaling [56]. In HG3 cells, the expression of Mcl-1 following quercetin treatment (25 μM) was reduced of about 5-fold after 2 h of treatment and correlated with inhibition of the activating phosphorylation of Akt on Ser473 (Figure 2). It is worthwhile to note the extremely rapid effect of quercetin on Akt de-phosphorylation (3-fold decrease after 5 min), suggesting a fast uptake of the molecule and/or the presence of a substrate able to bind quercetin with high affinity.

### Quercetin uptake in HG3 cells

**Figure 3:**
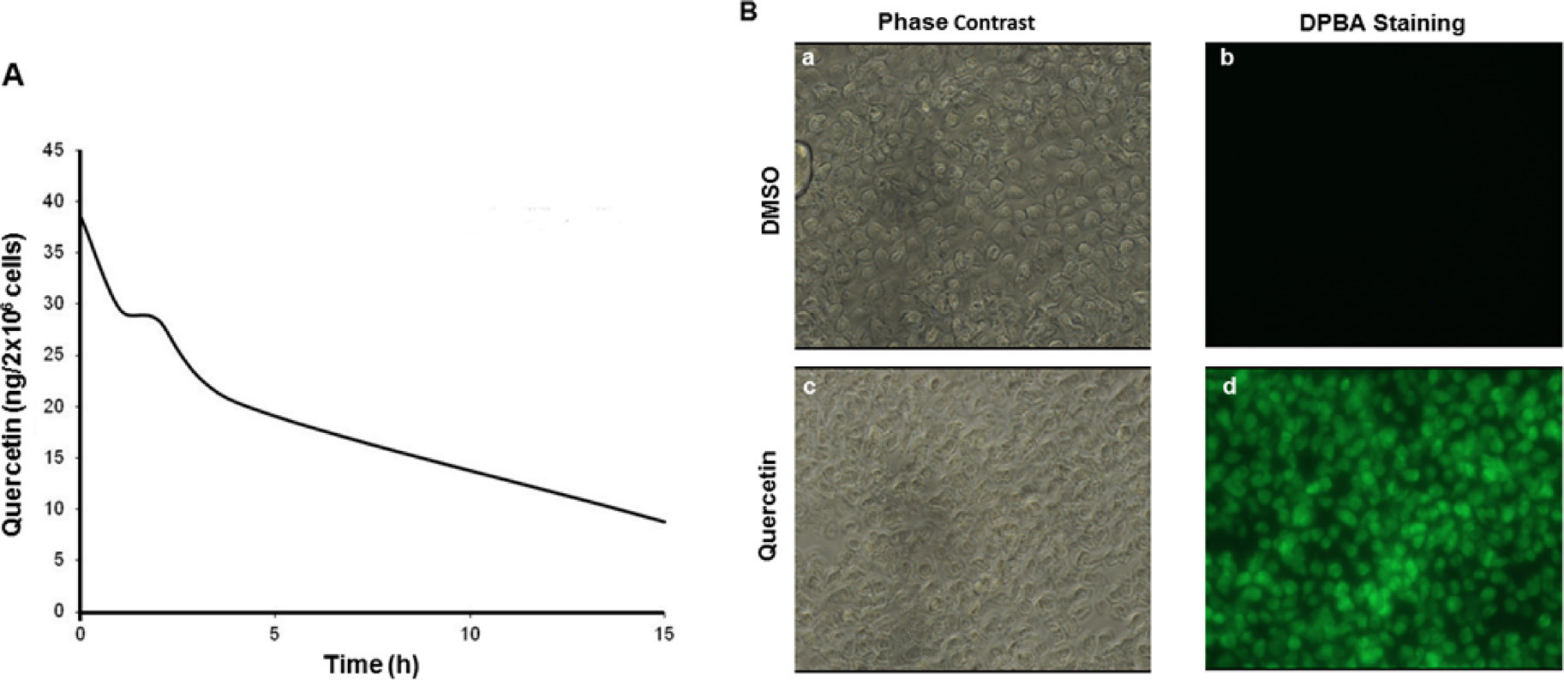
Quercetin stability in HG3 cells (**A**) Cells were incubated in the presence of 25 μM quercetin or vehicle control (DMSO) at different times (5, 60, 120, 240 min until 18 h); subsequently, quercetin was quantified in cells as described in Materials and Methods. (**B**) Cells (1 × 10^6^/ml) were treated with 25 μM quercetin or DMSO (0.1% v/v) before DPBA staining (5 min) performed as described in Materials and Methods. Cells were visualized using a fluorescent microscopy and photographed in phase contrast (a and c) and in FITC filter with 400× magnification (b and d).

To verify if quercetin was bioavailable in HG3, we treated cells with increasing concentrations of the molecule and measured its time-dependent incorporation. As reported in Table 1, quercetin was clearly measurable even at 5 min from treatment at all concentrations tested. Treatment with 25 μM quercetin resulted in an incorporation of 38.47 ± 16.46 ng/2 × 10^6^ cells, very shortly after its addition to the cell culture medium (5 min). The uptake depended upon concentrations applied and quercetin stability decreased over time. In fact, as reported in Figure 3A, quercetin decreased of about 4-fold after 15 h from treatment at 25 μM. The presence of quercetin in HG3 cells was also easily and clearly evidenced loading cells with DPBA, a dye which specifically binds flavonols (Figure 3B).

**Table 1:** Quercetin uptake in HG3 cell line

Control	Treatment (min)	Quercetin concentrations applied		
		5 μM	25 μM	50 μM
0.35 ± 0.03*	5	8.89 ± 1.04*	38.47 ± 16.46*	103.16 ± 15.72*
ND	60	7.54 ± 0.44*	29.52 ± 15.11*	79.67 ± 14.45*

*numbers indicate ng quercetin/2 × 10^6^ cells + s.d.

### PI_3_K enzymatic activity is directly inhibited by quercetin

**Figure 4:**
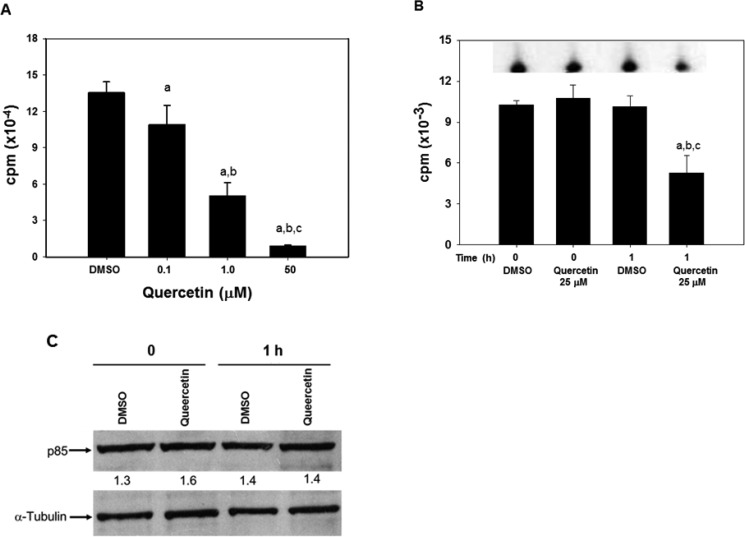
Quercetin inhibits PI_3_K activity *in vitro* and in HG3 cells (**A**) *In vitro* kinase assay was performed using a commercially available kit (Abcam) by measuring the amount of radioactively labeled [γ-^32^P] ATP incorporated into a lipid substrate, as described in the manufacturer's protocol in the presence of the range of quercetin concentrations indicated. The enzymatic activity was determined using as catalytic subunit the human recombinant PI_3_K-γ (His tagged) produced in sf9 insect cells and included in the kit. Symbols (a, b, c, d) indicate significance with respect to DMSO (a) and treated cells (b = 0.1 μM quercetin; c = 1 μM quercetin; d = 50 μM quercetin); *p* < 0.01 for all determinations, except for a versus b where *p <* 0.05 (one-way ANOVA test). (**B**) Cells (5 × 10^6^/ml) were incubated in the presence of 25 μM quercetin, or DMSO (0.1% v/v) as control, for the indicated time (0 and 1 h). Immunoprecipitation was performed as reported in Materials and Methods section on 500 μg of total proteins using an antibody reacting against the p85-α and -β regulatory subunits of class I PI_3_Ks. The immunoprecipitates were used as enzymatic source to detect PI_3_K activity using the commercial kit described in panel A. The insert on top of the graph shows a representative autoradiogram of ^32^P-phosphorylated lipid substrate, following separation using TLC, as reported in Materials and Methods. Symbols (a, b, c) indicate significance; *p* < 0.05 with respect to DMSO t = 0 (a); DMSO t = 1 h (c) and treated cells (b = 25 μM quercetin at t = 0; d = 25 μM quercetin at t = 1 h) (one-way ANOVA test). (**C**). After the kinase assay, the same immunoprecipitates used in panel B were washed to eliminate the reaction buffer remaining after the kinase reactions, added with loading buffer, boiled for 5 minutes and loaded on a 4–12% pre-cast gel before immunoblotting. The membrane was incubated with primary antibody against p85-α and -β regulatory subunits of class I PI_3_Ks, or α-tubulin. Band intensities were quantified measuring optical density on Gel Doc 2000 and analysed by Multi-Analyst Software. Numbers between the panels indicate p85 expression normalized respect to α-tubulin. The immunoblot is representative of at least two independent experiments.

Based on the previous results, we hypothesized that PI_3_K could represent an early target of quercetin in HG3. This conclusion was based on several experimental evidence: i. the fast uptake of quercetin (Table 1 and Figure 3); ii. the rapid deactivation of Akt (Figure 2); iii. the capacity of quercetin to target the ATP binding of PI_3_K and inhibit its enzymatic activity [57, 58]. To confirm this hypothesis, we firstly demonstrated that quercetin was able to inhibit the recombinant catalytic subunit of PI_3_K, with an IC_50_ of about 14 μM (Figure 4A). This assay was performed *in vitro* employing the recombinant enzyme present in the commercially available PI_3_K assay kit (see Methods section). Subsequently, we immunoprecipitated PI_3_K from quercetin treated HG3 cells using an antibody able to recognize the p85-α and -β regulatory subunits of class I PI_3_Ks. As reported in Figure 4B, treatment with 25 μM quercetin reduced of about 50% the immunoprecipitated enzymatic activity of the *bona fide* PI_3_K isoforms expressed in HG3 cells, after 1 h of treatment. The decreased enzymatic activity was not due to a reduced expression or amount of the immunoprecipitated kinase, since the immunoblot in Figure 4C does not show any significant changes in p85 levels, confirming the capacity of quercetin to inhibit PI_3_K enzymatic activity.

### CK2 enzymatic activity is directly inhibited by quercetin upstream of PI_3_K

**Figure 5:**
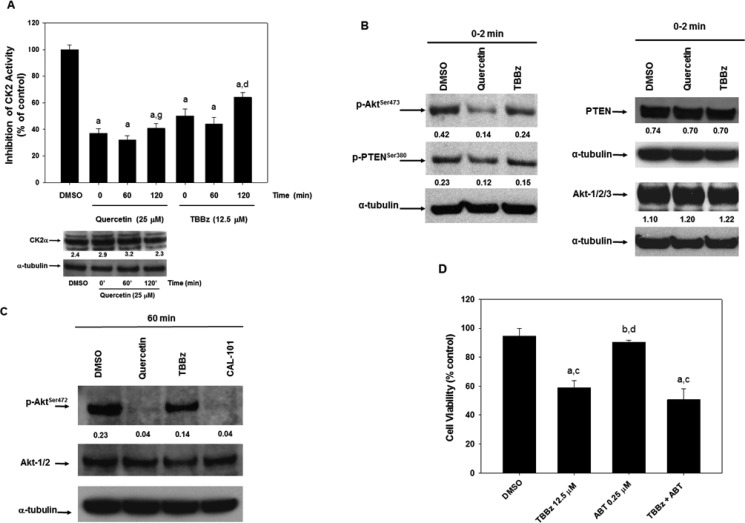
Quercetin inhibits CK2 activity restoring the control of PTEN on PI_3_K-Akt pathway (**A**) HG3 cells, after treatment with 0.1% DMSO, 25 μM quercetin, 12.5 μM TBBz for the indicated times, were lysed and used to assay CK2 activity as described in Materials and Methods. Symbols (a, d) indicate significance with respect to DMSO (a) and treated cells (d = 25 μM quercetin at t = 120 min; g = 12.5 μM TBBz at t = 120 min); *p* < 0.001 for all determinations, except for d versus g where *p <* 0.05 (one-way ANOVA test). The immunoblot on the bottom of the panel shows the expression of CK2α subunit detected using a not commercial, anti-CK2α antibody. The membrane was re-probed with the anti α-tubulin antibody. Band intensities were quantified measuring optical density on Gel Doc 2000 and analysed by Multi-Analyst Software. Numbers between the panels indicate CK2α expression normalized respect to α-tubulin. The image is representative of one out of two experiments performed. (**B**) and (**C**) HG3 cells were treated as in panel A for 0–2 min (B) or 60 min (C). After immunoblotting, the membranes were incubated for 16 h at 4°C with anti-phospho-Akt (p-Akt^Ser473^), anti-phospho-PTEN (p-PTEN^Ser380^), anti-Akt1/2/3, anti-PTEN, or α-tubulin antibodies. In panel C, the treatment with CAL-101 (5 μM) was also included. Band intensities were quantified measuring optical density on Gel Doc 2000 and analysed by Multi-Analyst Software. In panel B, numbers between panels indicate the expression of p-Akt^Ser473^ and p-PTEN^Ser380^, Akt1/2/3 and PTEN normalized respect to α-tubulin. In panel C, numbers between top and middle panels indicate the expression of p-Akt^Ser473^ normalized respect to Akt1/2/3. Immunoblots are representative of at least three independent experiments performed. (**D**) HG3 cells were treated at the concentrations indicated for 24 h and the effect of TBBz, ABT-737 and their combination on cell viability (neutral red assay) was measured. Symbols (a, b, c, d) indicate significance with respect to DMSO (a) and treated cells (b = 12.5 μM TBBz; c = 0.25 μM ABT-737; d = TBBz+ABT-737); *p* < 0.001 (one-way ANOVA test).

The observation that the uptake of quercetin and the dephosphorylation of Akt “preceded” the inhibition of PI_3_K, suggested the possibility that an upstream modulator of PI_3_K/Akt pathway could be also targeted by quercetin. It is known that PTEN is a negative regulator of PI_3_K [59] and CK2 phosphorylates PTEN on several residues inducing its functional inhibition [60]. In addition, we and others previously reported that pure CK2 enzyme can be competitively inhibited by quercetin [61]. As reported in Figure 5A, CK2 activity was inhibited in HG3 cells treated with 25 μM quercetin, soon after addition of the flavonol (in the range of 0–2 min, indicated as 0 min in Figure 5A). The inhibition persisted for several hours and the inhibitory effect of quercetin treatment was superimposable to the effect of a CK2 specific inhibitor, TBBz [62]. The immunoblot on the bottom of Figure 5A shows that the expression of CK2α does not change significantly upon treatment with quercetin, confirming that the decreased kinase activity was due to quercetin inhibition, not to changes in the expression of the catalytic subunit. The consequence of CK2 inhibition by quercetin on the PI_3_K-Akt pathway was confirmed by the immunoblot in Figure 5B which shows PTEN dephosphorylation on Ser380, one of the phosphorylation site triggered by CK2, at 2–5 min following both quercetin and TBBz treatments. As expected, PTEN dephosphorylation coincided with Akt inactivation at the same experimental times. Also in this case, the control immunoblottings on the right side of Figure 5B show that changes in phospho-PTEN and phospho-Akt levels were not due to reduced expression of the total intracellular PTEN and Akt proteins. It is worthwhile to note that comparing the different times of quercetin and TBBz treatments (5 versus 60 min), the capacity of TBBz to reduce the activating Akt phosphorylation on Ser473 significantly decreased at 60 min (Figure 5C) compared to 5 min (Figure 5B). On the contrary, inactivation of Akt by quercetin increased at 60 min and was superimposable to CAL-101 (Figure 5C), a well-known and specific inhibitor of PI_3_K-Akt pathway. Finally, we demonstrated that TBBz was unable to complement quercetin in the association with ABT-737, resulting in increased sensitivity to apoptosis. In fact, Figure 5D shows that the effect of TBBz plus ABT-737 on cell viability was not synergic, but barely additive. This was also confirmed by the calculation of the C.I. which remained close to 1 (data not shown).

### Quercetin is selective for BH3-mimetic compound triggering Bcl-2/Bcl-X_L_

**Figure 6:**
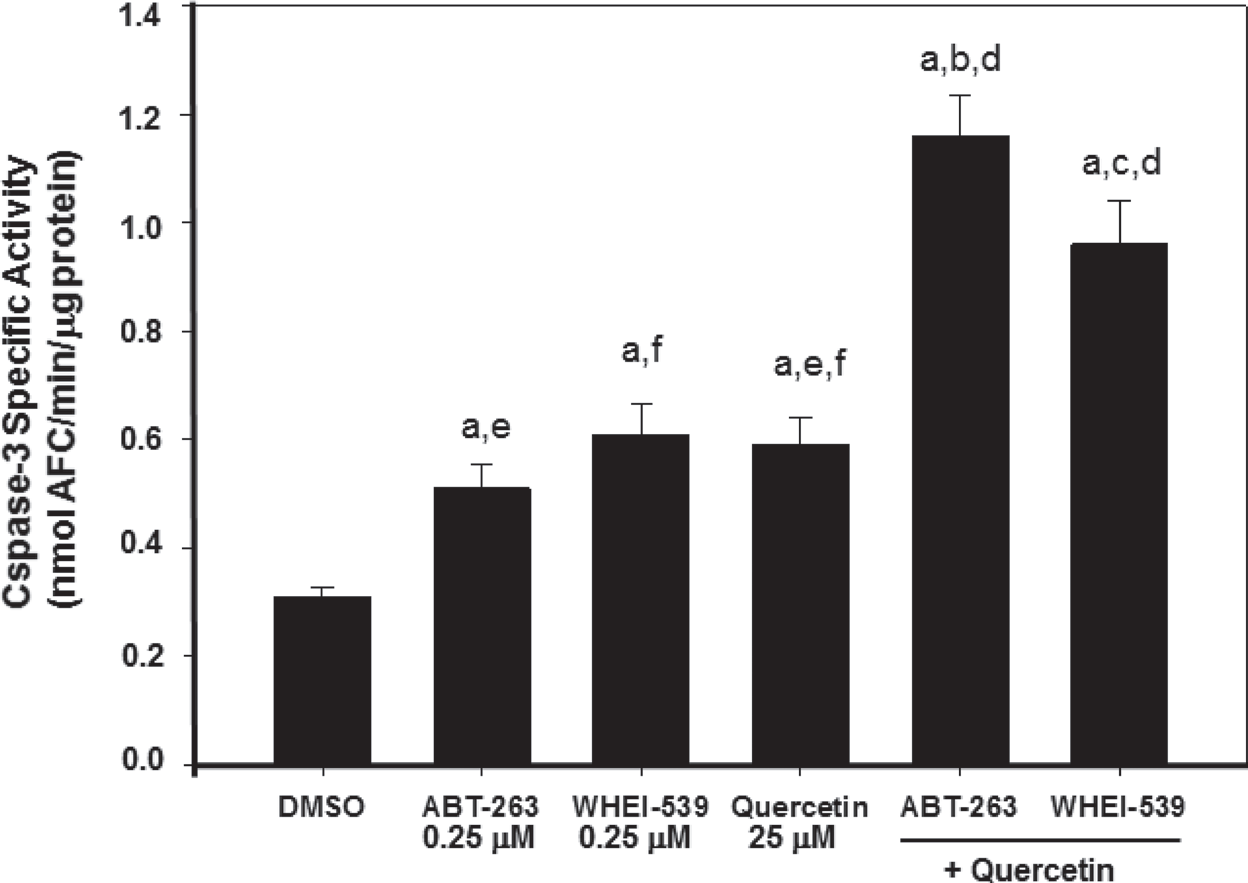
Quercetin and BH3-mimetics enhance caspase-3 activity in HG3 cells Activation of caspase-3 was measured after 6 h of incubation with the indicated reagents (0.25 μM ABT-263, 0.25 μM Whei, 25 μM quercetin) using the enzymatic assay described in Materials and Methods. Specific caspase-3 activity was expressed as nmol AFC/min/μg protein. Symbols (a, b, c, d, f, g) indicate significance with respect to DMSO (a) and treated cells (b = 0.25 μM ABT-263; c = 0.25 μM WHEI-539; d = 25 μM quercetin; e = 0.25 μM ABT-263 + 25 μM quercetin; f = 0.25 μM WHEI-539 + 25 μM quercetin); *p* < 0.001 for all determinations except for a versus b where *p <* 0.05 (one-way ANOVA test).

We reasoned that quercetin efficacy in restoring sensitivity to the apoptotic effect of ABT-737 was dependent upon destabilization of Mcl-1, which is not a specific target of ABT-737. However, ABT-737 is essential to target and inactivate Bcl-2 and Bcl-X_L_. Therefore, using BH3-mimetics with limited affinity for Mcl-1, but able to selectively inhibit the pro-survival functions of Bcl-2 and Bcl-X_L_, we expected that these compounds would behave similarly to ABT-737 plus quercetin. In fact, when ABT-263 (Navitoclax) and Wehi-539, which bind with higher efficiency than ABT-737 to Bcl-2 and Bcl-X_L_, respectively [63, 64], were associated with quercetin in HG3 cells, they showed a synergistic effect on apoptotic induction, comparable to the co-treatment ABT-737 plus quercetin. Table 2 reports the calculation of the C.I. for the two combinations ABT-263 plus quercetin and Wehi-539 plus quercetin. In both cases, the C.I. resulted < 1 and in the same range of those reported in Figure 1B for ABT-737 and quercetin. Increased apoptosis, determined by measuring the activation of caspase-3, was detected in all co-treatments investigated (Figure 6).

**Table 2:** C.I. values for different BH3-mimetics when associated with quercetin in HG3 cell line

	Total dose*	Fa	C.I.
**ABT-236/Quercetin [1/80]**	10.12	0.58	0.368
	20.25	0.72	0.364
**WHEI-539/Quercetin [1/80]**	10.12	0.23	0.465
	20.25	0.35	0.310

As a corollary of the results obtained with ABT-263 and Wehi-539, we observed that quercetin loses its synergistic capacity when associated with TW-37, a BH3- mimetic compound with higher affinity for Mcl-1 [65]. In fact, the calculated C.I. value indicated only an additive effect (even antagonist at high Fa) and the apoptotic capacity of quercetin plus TW-37 was also strongly reduced (data not shown).

## DISCUSSION

In the Introduction, we reported that one of the possible approaches against CLL is the design of new therapeutic strategies to bypass the acquired resistance to apoptosis occurring in B-cells. This goal can be achieved with the recurrence to a new generation of BH3-mimetics compounds, Venetoclax is an example [27]. Alternatively, novel protocols can be applied, based on the combination of chemotherapy and immunotherapy in relation to the Binet or Rai stages of the disease, the physical fit of the patients, the presence of a del(17p) or TP53 mutation (reviewed in [66, 67]). As an example, in the latter situation, significant improvements in terms of progression-free survival, response rate and overall survival have been obtained with the combination of Idelalisib (formerly known as CAL-101 or GS-1101), a first-in-class inhibitor of the PI_3_K delta isoform, in combination with rituximab [68]. The case of Idelalisib further highlights the importance of inhibiting the PI_3_K-Akt pathway for an efficient therapy against CLL.

The present article reinforces the notion that plant-derived compounds can enhance apoptosis in CLL, as extensively reviewed elsewhere [15, 69]. However, we added new contributions: 1. quercetin represents one of the few compounds (if not the unique) where its direct molecular target(s) in CLL-derived cells have been identified; 2. quercetin requires the presence of specific BH3-mimetics compounds to “synergistically” enhance apoptosis, at least in *in vitro* and *ex vivo* models. In the next paragraphs, we will deeply discuss these two issues.

**Figure 7:**
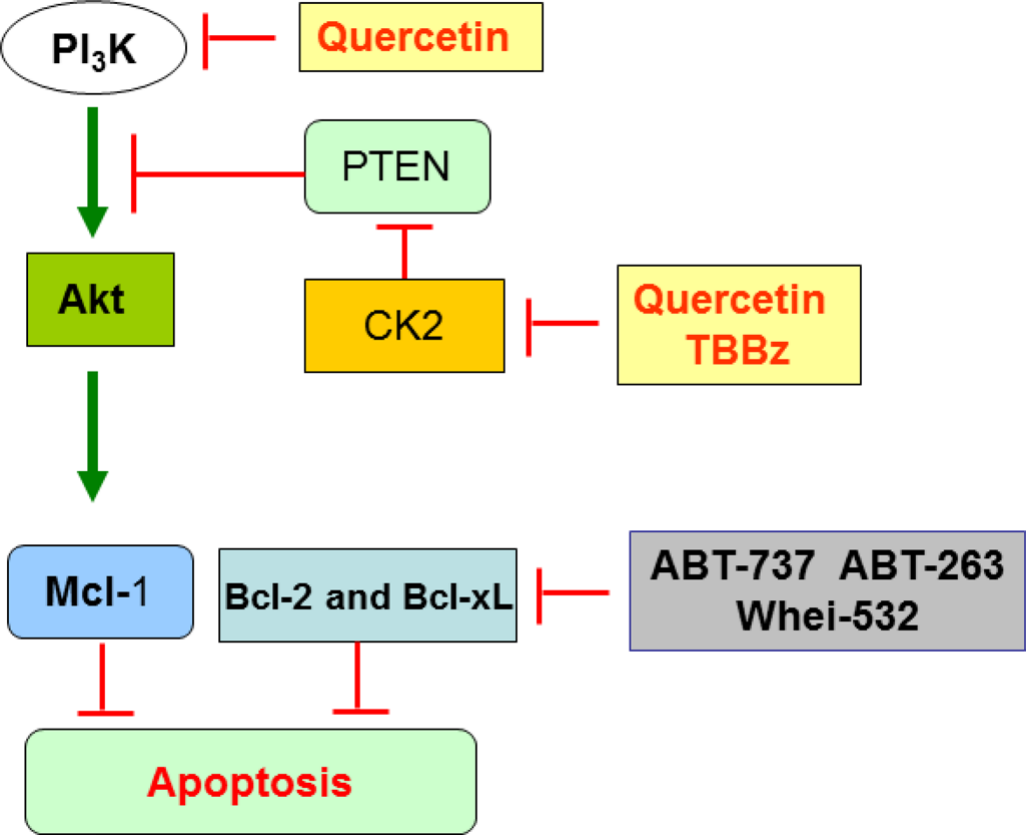
Scheme summarizing the key targets of quercetin in HG3 cells The double inhibitory activity of quercetin on CK2 and PI_3_K converges on Akt pathway, which is inactivated and unable to phosphorylate and stabilize anti-apoptotic Mcl-1. In parallel, ABT-737 (or other BH3-mimetics) can trigger and inhibit different anti-apoptotic members of Bcl-2 family. The combined effect of quercetin plus ABT-737 restores sensitivity to apoptosis in HG3 and CLL (see text for details).

Quercetin ability to act as “not specific” inhibitor of serine-threonine kinases, with a K_i_ in the micromolar range, is known since the late nineties [70]. We and others demonstrated that quercetin directly binds and inhibits recombinant or purified forms of catalytically active CK2 and PI_3_K; this inhibition occurs also on the cellular enzymes (this paper; [58, 61, 71]). Here, we focused on the “timing” of quercetin effects, being CK2 enzymatic activity inhibited “immediately” after the cellular uptake of the flavonoid, while PI_3_K was inhibited later, but, in any case, within 1 h from quercetin treatment. This represents an important novelty in considering the multifunctional effects of naturally occurring compounds, whose capacity to inhibit cell growth has been often erroneously defined without carefully considering: i. their active intracellular concentration; ii. their specific molecular target(s); iii. their “affinity” for one pathway over others that can also be triggered, but with less specificity or at later times. The demonstration that CK2, and, consequently, the CK2/PTEN pathway (Figure 7), represents the primary target of quercetin in HG3 cells, being inhibited at time 0–2 min from treatment, does not mean that the same mechanism occurs in other cellular systems, where quercetin may show higher affinity for other substrates/pathways besides CK2/PTEN. More in general, our finding opens the road to re-evaluate the specificity of plant-derived compounds in cancer cells.

Regarding quercetin binding and inhibition of CK2 activity, strong structural evidence suggest that quercetin competes with the ATP binding site. Elegant experiments addressed this issue. The planarity of the molecule, pre flavone scaffold with at least two hydroxyl groups at positions 7 and 4′ ensure the competitive inhibition with respect to ATP [72]. Using a different approach, a biotinylated version of quercetin has been employed as intrinsic photo-affinity proteomics probe to capture CK2 as substrate from both an *in vitro* pull-down experiments on the pure enzyme and from CK2 present in cellular extracts. In this case, it has been proposed that quercetin may bind to the allosteric cleft between α/β surface of CK2, an interacting region between α, β subunits crucial to the function of CK2 holoenzyme [73]. Based on this evidence, it is plausible that similar inhibition mechanisms are active *in vivo* to explain the rapid inactivation of CK2 observed in HG3 cells following quercetin treatment.

Regarding the inhibition of PI_3_K by quercetin, it is worthwhile to mention that X-ray crystallographic structure of PI_3_K-γ isoform bound to quercetin indicates that the molecule fits into the ATP binding site of the kinase with a K_*d*_ value of 0.28 μM. It is also interesting that the orientation of quercetin into the ATP binding site is different compared to myricetin, a quercetin analog which only differs for the addition of a hydroxyl at the 5′-OH of the phenyl moiety, and LY294002, a specific PI_3_K inhibitor whose structure has been designed using quercetin as lead compound [58]. This observation indicates a possible specificity of quercetin compared to other PI_3_K inhibitors. Probably, the same competitive enzymatic inhibition of PI_3_K isoforms occurs *in vivo* to explain the results shown in Figure 4. We do not know exactly which PI_3_K isoform(s) were present in the immunoprecipitation shown in Figure 4B, since we employed an antibody which recognizes the p85 regulatory subunit of class IA PI_3_K enzymes, able to bind the catalytic subunits p110-α, -β and –δ. However, considering that class I PI_3_Ks are present in all cell types, with p110–δ and –γ enriched in leukocytes and B-CLL [59, 74], we can *bona fide* conclude that PI_3_K-δ is present in HG3 cells, as confirmed by the observation that the treatment with CAL-101(Idelasib) resulted in a strong reduction of Akt phosphorylated on the activating residue Ser473 (Figure 5C).

As schematically summarized in the cartoon shown in Figure 7, the double inhibitory activity of quercetin on CK2 and PI_3_K converges on Akt, which is inactivated and unable to phosphorylate and stabilize anti-apoptotic Mcl-1 [75]. In parallel, ABT-737 (or other BH3-mimetics) can trigger and inhibit different anti-apoptotic members of Bcl-2 family. The combined effect of quercetin plus ABT-737 restores sensitivity to apoptosis in HG3 and possibly CLL. The present model raises several spontaneous questions: i. what is the advantage to use quercetin over more specific kinase inhibitors for CK2 and/or PI_3_K, such as TBBz and CAL-101, respectively? ii. why does the combined treatment of quercetin plus ABT-737 generate a synergistic effect instead of an additive one? About the first issue, theoretically, quercetin allows “to hit two birds with one stone”, since the molecule inhibits two signaling pathways, both converging on Akt inactivation and Mcl-1 destabilization. The use of a single compound (quercetin), instead of the combination of two (TBBz + CAL-101), is useful in reducing toxicity and, generally, unwanted side effects. As shown in Figure 5C, quercetin efficacy in reducing phospho-Akt^Ser473^ was comparable to CAL-101. In addition, the combination ABT-737 plus TBBz reduced cell viability additively compared to the synergistic effect of ABT-737 plus quercetin (consider Figure 1 versus Figure 5D). This observation opens the question on why and how the latter treatment is synergistic. Several studies report that understanding synergism may explain the mechanism of action of even a single agent [76]. In the case of quercetin and ABT-737, to be synergic, we can image that the two pathways, respectively triggered by these compounds, must converge on a common, downstream target which, in turns, amplifies the apoptotic signal. Currently, this hypothesis is not supported by strong experimental evidence. As an alternative explanation, we can image that quercetin triggers other substrates involved in the resistance to apoptosis, lowering the threshold necessary to favor a pro-apoptotic response. Three circumstantial evidence support this view: i. we previously demonstrated that in U-937 cells, quercetin downregulates Mcl-1 acting directly or indirectly on its mRNA stability and protein degradation.[32]; ii. similarly to other phytochemicals [77], quercetin may up-regulate NOXA which is important in CLL since the ratio NOXA/Mcl-1 dictates CLL sensitivity to ABT-737 [78] (Russo M. et al., unpublished); iii. recently, it has been described the capacity of quercetin to directly bind the BH3 domain of Bcl-2 and Bcl-X_L_ proteins, inhibiting their activity and promoting cancer cell apoptosis. Structural similarities seem to exist in the interactions between the Bcl-X_L_/quercetin compared to Bcl-X_L_/ABT-737 complexes, showing that quercetin can bind to Bcl-X_L_ as ABT-737 [79]. This latter approach, although largely based on *in vitro* data, opens new perspectives to the interpretation of the mechanism of action of quercetin in B-CLL and deserves further investigations.

Is it possible to consider a clinical outcome of quercetin, in combination with BH3- mimetic antagonists? In the case of a therapeutic use of quercetin against CLL, its intravenously administration in patients undergoing chemotherapeutic treatment will ensure high circulating concentrations of the free aglycone and avoid the formation of conjugates. In this respect, a unique phase I clinical trial of quercetin has been so far completed, suggesting a recommended a dose of 1400 mg m^−2^, which corresponds to about 2.5 g for a 70 kg individual, administered via intravenous infusion at three-weeks or weekly intervals [80]. In addition, if quercetin becomes a molecule of pharmacological interest in CLL and/or other forms of cancer, its chemical bioavailability can be improved using different biotechnological approaches. To this aim, the recent literature is extremely fertile on proposing engineered formulations (e.g., silica, chitosan, PLGA and PLA nanoparticles, liposomes, micelles, etc.) of quercetin [81, 82]. Of course, these approaches require not only an enhanced target specific delivery of the nanoformulations, but also a high level of specificity towards cancer cells to prevent unwanted side effects on normal cells.

In conclusion, this study represents one of the few examples in the literature where the direct targets of quercetin in a specific form of cancer have been identified. It also highlights the importance to evaluate the intracellular uptake and stability of the molecule and to identify the earlier events related to its biological activity. This preclinical study may open new perspectives for a clinical trial on CLL patients where quercetin can be included in the chemotherapeutic protocol as adjuvant agent.

## MATERIALS AND METHODS

### Reagents

Roswell Park Medium Institute (RPMI) medium, L-glutamine 200 mM, penicillin 5000 IU/ml/streptomycin 5000 μg/ml and PBS (phosphate buffer saline) tablets were purchased from Invitrogen (S. Giuliano Milanese, MI, Italy); fetal bovine serum from Cambrex (Milano, Italy). Neutral red solution (0.33% v/v), trypan blue solution (0.4% v/v, propidium iodide, quercetin, TBBz (4, 5, 6, 7-tetrabromobenzimidazole) and dimetylsulfoxide (DMSO) were from Sigma-Aldrich (Milano, Italy). Cal-101 (GS1101) was purchased from Sellechem (Aurogene, Rome, Italy). ABT-737, ABT-263, Whei-532 and TW-32 7 were kindly provided by Apex-Bio (USA) and dissolved in DMSO, aliquoted and stored at −20°C. Methanol and formic acid HPLC grade were obtained from Merck (Vimodrone, Milano, Italy). HPLC grade water (18.2 MΩ) was prepared by using a Millipore Milli-Q purification system (Merck-Millipore).

### Cell culture, cell viability assay and apoptotic nuclei staining

HG3 cells, a lymphoblastoid cell line with B1 cell characteristics established from a chronic lymphocytic leukemia clone by *in vitro* EBV infection, were cultured in RPMI medium supplemented with 10%, fetal bovine serum, 1% L-glutamine and 1% penicillin/streptomycin at 37°C in a humidified atmosphere containing 5% CO_2_ [45].

Cell viability was assayed using neutral red viability test [83]. Cells were cultured at density of 0.25–0.5 × 10^6^/ml in 48 multi-well plates and incubated (24 h) in a medium containing the indicated compounds. Cell viability assay was performed as described [36]. Briefly neutral red (0.066% v/v final concentration) was added and incubated for 2 h. Cells were collected and centrifuged at 400 × *g* for 5 minutes, washed once with PBS and incubated with lysis buffer (50 mM Tris-HCl pH 7.4; 150 mM NaCl; 5 mM dithiotreitol; 1% Triton-100) containing 1% acetic acid and 50% ethanol. Absorbance was spectrophotometrically measured at 540 nm (Synergy HT multi-well reader; Bio-Tek Instruments, Milano, Italy). The quantity of adsorbed dye was proportional to number of living cells and/or with an active metabolism.

Combination index (C.I.) values were calculated according to the Chou and Talalay mathematical model for drug interactions [84], as previously reported [31]. Dose–response curves, dose–effect analysis and C.I. for the combination treatment groups were generated using the equations reported by Chou and Talalay using the CompuSyn software (freely available at: www.combosyn.com).

### Caspase assays

To determine caspase-3 enzymatic activity, 2 × 10^6^/ml cells were treated as indicated for 6 h. Subsequently, cells were collected and centrifuged at 400 × *g* for 5 minutes, washed twice in PBS and suspended in lysis buffer (10 mM Hepes, pH 7.4; 2 mM ethylenediaminetetraacetic acid; 0.1% [3-(3-cholamidopropyl) dimethylammonio]-1-propanesulfonate, 5 mM dithiothreitol, 1 mM phenylmethylsulfonylfluoride, 10 μg/ml pepstatin-A, 10 μg/ml apronitin, 20 μg/ml leupeptin). Cell extracts (10 μg) were added with the reaction buffer and the conjugated amino-4-trifluoromethyl coumarin (AFC) substrate: benzyloxycarbonyl-Asp (OMe)-Glu (OMe)-Val-Asp (OMe)-AFC (Z-DEVD-AFC). The samples were incubated at 37°C for 30 minutes. Upon proteolytic cleavage of the substrate by caspase-3, the free fluorochrome AFC was detected by a spectrofluorometer multiplate reader (Bio-Tek Instruments) with excitation 400 ± 20 nm and emission at 530 ± 20 nm. To quantify enzymatic activity, we determined an AFC standard curve. Caspase-3 specific activity was calculated as nmol of AFC produced per min per μg proteins at 37°C in the presence of saturating substrate concentration (50 μM) [36].

### Annexin V assay

Phosphatidylserine exposure was measured using the binding of fluorescein-isothiocyanate-labeled (FITC) Annexin V to phosphatidylserine, as indicated in the manufacturer's protocol (Milteny-Biotech, Milano, Italy). Briefly, cells (1 × 10^6^/ml) were treated as indicated for 16–18 h (over-night). Cells were collected and centrifuged at 400 × *g* for 5 minutes, washed in PBS and suspended in binding buffer (10 mM Hepes, pH 7.4; 140 mM NaCl; 2.5 mM CaCl_2_). Annexin V FITC (10 μL) and propidium iodide (25 μg/ml) were added to the cells for 10 min in the dark at room temperature and analyzed with flow cytometer (FACSCalibur; Becton Dickinson, Mountain View, CA, USA) equipped with argon laser (488 nm) and filtered at 530 and 585 nm for FITC and phycoerythrin respectively. Low fluorescence debris and necrotic cells, permeable to propidium iodide, were gated out before to analysis. Data were analyzed using CellQuest software (Becton Dickinson).

### Immunoblot

After treatments, cells (generally 1.5 × 10^6^) were suspended in lysis buffer containing 50 mM Tris/HCl, pH 7.4; 150 mM NaCl; 5 mM ethylenediaminetetraacetic acid; 1% Nonidet P-40; 0.5 mM dithiotreitol; 1 mM Na_3_VO_4_; 40 mM NaF; 1 mM Na_4_P_2_O_7_; 7.4 mg/ml 4-p-nitrophenyl phosphate; 10% glycerol; 100 μg/ml phenylmethylsulfonyl fluoride and the cocktail of protease inhibitors ‘complete’ (Roche Applied Science; Monza, Italy). Following measurement of protein concentration [85], total lysates (20–25 μg) were added with loading buffer (Bio-Rad Laboratories, Milano, Italy), boiled for 5 minutes and loaded on a 12% pre-cast gel (CRITERION XT; Bio-Rad Laboratories) using MOPS [3-(N-morfolin) propanosulfonic] buffer (1 M MOPS; 1 M Tris/Base; 69.3 mM SDS; 20.5 mM EDTA) or MES [2-(*N*-morpholino)ethanesulfonic acid] buffer (50 mM MES, 50 mM Tris/Base, 0.1% SDS, 1 mM EDTA) and a constant voltage (200 V). Proteins were blotted onto polyvinylidene difluoride (PVDF), membrane (Transfer Pack Bio-Rad Laboratories), using TRANS-Blot TURBO System (Bio-Rad laboratories), with a constant amperage (2.5 mA) for 7 min room temperature. The membranes were rinsed with T-TBS (0.1% Tween-20; 25 mM Tris; 137 mM NaCl; 2.69 mM KCl, pH 8) and blocked using 5% (w/v) non-fat dry milk resuspended in T-TBS for 1 h at room temperature, before incubation for 16 h at 4°C with specific antibodies. The primary antibodies used were anti-Mcl-1, anti-pAKT, anti pPTEN (Cell Signaling; Milano; Italy), anti-Caspase-3 (Genetex), anti-p85-α and -β regulatory subunits of class I PI_3_Ks (Santa Cruz Biotechnologies, Heidelberg, Germany), anti-Akt1/2/3 (Santa-Cruz Biotechnologies; kindly provided by Dr. Paola Ungaro), anti-PTEN (Upstate Biotechnology; kindly provided by Prof. Adriana Borriello), anti-CK2α subunit (Ab 276 which recognizes α–α′ subunits, [86]), anti-α-tubulin (Sigma-Aldrich). PVDF membranes were finally incubated with horseradish peroxidase linked secondary antibody against mouse or rabbit (GE Healthcare, Milano, Italy) and immunoblots developed using the ECL Plus Western Blotting Detection System Kit (Perkin-Elmer, Milano, Italy). Band intensities were quantified measuring optical density on Gel Doc 2000 Apparatus and Multi-Analyst Software (Bio-Rad Laboratories).

### Kinase assays

PI_3_K activity was determined using a commercially available kit (Abcam, Cambridge, UK) by measuring the amount of radioactively labeled [γ-32 P] ATP incorporated into a lipid substrate, following separation using thin layer chromatography (TLC), as described in the manufacturer's protocol. The enzymatic activity was determined using the pure enzyme, i.d. the PI_3_K recombinant catalytic subunit included in the kit, or the active enzyme immunoprecipitated from HG3 cells treated with quercetin as indicated in figure legends. The immunoprecipitation was performed using an antibody reacting against the p85-α and -β regulatory subunits of class I PI_3_Ks (Santa Cruz Biotechnology; cat. # sc-423) following a previously described protocol [87].

CK2 activity was determined on cell lysates deriving from DMSO (0.1%, v/v) and HG3 treated cells with different reagents (25 μM quercetin, 12.5 μM TBBz, as indicated in figure legends). The reaction mixture contained in a total volume of 50 μl, 50–90 μM [γ-32 P] ATP (1500–3000 cpm/pmol ATP) (Perkin-Elmer), 0.5 mM peptide substrate ETE (RRREEETEEE, amino acid sequence one letter code; Promega, Milan, Italy) in CK2 kinase buffer (50 mM MOPS, pH 7.0, 10 mM MgCl2, 10 mM NaCl, 60 mM β-glycerophosphate), as previously reported [40, 86]. Reactions were incubated for 30–40 min at 30°C in the presence of 5–10 μg of cell lysate and terminated by transferring the supernatants to P81 paper (Whatman; GE Healthcare) to determine phosphate incorporation on ETE peptide as described [88].

### Measurement of intracellular levels of quercetin

To detect the intracellular levels of quercetin, HG3 cells (1.5–2.0 × 10^6^) were incubated in the presence of different concentrations of quercetin (5–50 μM) in RPMI containing 10% FBS, 1% L-glutamine, and 1% penicillin/streptomycin. At the end of incubation (5–60 min), cells were harvested by centrifugation and washed two times in PBS. Cellular pellets were re-suspended three times with methanol and sonicated. Finally, supernatants were frozen at −80°C, until HPLC analysis was performed. Quercetin concentrations in extracts were determined by HPLC–UV analysis using a HP 1110 series HPLC (Agilent, Palo Alto, CA, USA) equipped with a binary pump (G-1312A) and a UV detector (G-1314A). Samples were analyzed using a reverse phase Hypersil BDS C18 column (250 mm 4.6 mm, 5μm) (Thermo, Bellefonte, PA, USA) at a flow rate of 1 ml min^−1^. Solvent A was 0,1% formic acid and solvent B was 0.1% formic acid in acetonitrile. The gradient for B was as follows: 30% for 5 min, from 30% to 95% in 20 min, from 95% to 100% in 2 min. The eluate was monitored at 380 nm. In order to confirm the identity of quercetin HPLC eluted peaks were analyzed by Electrospray Ionization multistage Ion Trap Mass Spectrometry (ESI-ITMS^n^) using a Finnigan LCQ DECA XP Max ion trap mass spectrometer (Thermo Finnigan, San Josè, CA, USA), equipped with Xcalibur^®^ system manager data acquisition software (Thermo Finnigan, San José, CA, USA). Mass spectra were recorded from mass-to-charge ratio (m/z) 80 to 600 in negative ionization mode. The capillary voltage was set at −10 V, the spray voltage was at 3 kV and the tube lens offset was at −10 V. The capillary temperature was 275°C. Data were acquired in MS, MS/MS and MS^n^ scanning mode. Quantification of quercetin was performed with external calibration curves generated by repeated injections of a fixed volume of quercetin standard over a concentration range of 0.01 a 0.2 μg μl^−1^, with five different concentrations and duplicate injections at each level. All samples were prepared and analyzed in duplicate. Experiments were performed three times in duplicates and expressed as ng quercetin/2 × 10^6^ cells. Table 1 represents the mean of three experiments (± s.d).

### DPBA staining

To qualitatively detect quercetin uptake in HG3 cells, we used DPBA (2-aminoethyl diphenylborinate; Sigma-Aldrich) staining, a reagent used in plant physiology that selectively binds flavonols emitting fluorescent when exited at 485 ± 20 nm (emission at 530 ± 20 nm) [89]. The method was slightly modified to qualitatively assess the intracellular presence of quercetin in human cell lines. Briefly, HG3 cells were plated at a cell density of 1 × 10^6^/ml in a 12-wells plate and treated with 25 μM quercetin (5–60 min); subsequently, cells were centrifuged, washed with PBS, suspended in 1 ml of DPBA solution (2.5 mg/ml) dissolved in 10% formalin and finally incubated at 37°C in a humidified atmosphere containing 5% CO_2_ for 15 min. After incubation, cells were visualized using a fluorescent microscopy and photographed in phase contrast and in FITC filter with 400× magnification (Axiovert 200 Zeiss, Iena Germany).

### Statistical analysis

Statistical analyses were performed using IBM SPSS Statistics (version 23.0.; IBM Corp). Results are expressed as mean ± standard deviation (s.d.). Differences between groups were tested using analysis of variance (ANOVA) with Bonferroni's correction for multiple comparisons. The significance was set at *p* < 0.05 with specific values indicated in figure legends.
